# Disability management in a public-private health care facility in South Africa: an organisational perspective

**DOI:** 10.4314/ahs.v21i3.55

**Published:** 2021-09

**Authors:** Rubendri Govender, Pragashnie Govender, Deshini Naidoo

**Affiliations:** 1 Department of Health, KZN, South Africa; 2 Discipline of Occupational Therapy, School of Health Sciences, University of KwaZulu-Natal, (Westville Campus), South Africa

**Keywords:** Disability management, vocational assessments, return to work

## Abstract

**Background:**

Job retention, long-term absenteeism and medical boarding pay-outs are significant concerns for employers within the public health care sector of South Africa.

**Objective:**

To describe disability management policies, procedures and programmes of employees following impairment and disability in a public-private healthcare facility in South Africa.

**Methods:**

An exploratory qualitative study design was used with key informants in senior management and nursing managers (n=12) selected via purposive sampling. Audio-recorded data from semi-structured interviews and a focus group were thematically analysed using inductive reasoning.

**Results:**

There is poor adherence to occupational health and disability management policies and the current referral process is informal with managers using discretion to manage employees with ill health and acquired disability. The procedures prescribed in the policy and procedure on incapacity and ill-health-retirement need to be followed, and an early return to work programme within the health care facility needs to be implemented.

**Conclusions:**

Despite South Africa having many policies on recruitment and reasonable accommodations, there is a lack of implementation of these policies. An integrated disability management policy and programme encompassing health prevention, early return to work strategies, vocational rehabilitation and the implementation of reasonable accommodation is required to ensure that employees who have acquired disabilities or ill health are successful in the workplace.

## Introduction

Job retention, long-term absenteeism and medical boarding pay-outs are major concerns for employers within the public health care sector in South Africa (SA)^1^. Within this sector in SA, disability equity is still a significant challenge. Despite labour laws emphasising that employers should accommodate employees with ill-health and acquired disabilities, there is still a greater focus on providing compensation and little focus on return to work (RTW) strategies and rehabilitation resulting in a decline in the employment of persons with disabilities (PWDs) within the labour force^2^,^3^,^4^. During 2009, the Public Service Commission Report on Disability and Equity in the public service indicated that the within the KwaZulu-Natal (KZN) public service, the context in which this study occurred, the labour equity target of 2% for PWDs was not met with an employment rate of only 0,14%^5^,^6^. This despite the amended Employment Equity Act of 1998, protecting PWD against unfair discrimination and entitling PWD to affirmative action measures to facilitate their employment^2^.

Ill health, injury or disability acquired during employment appears to result in persistent long-term incapacity leave which affects personal, social and financial wellbeing of an individual^7^,^8^. According to the Policy and Procedure on Incapacity and Ill-health retirement (PILIR), “incapacity leave is additional sick leave granted conditionally at the employer's discretion, as provided for in PILIR”^7^,^8^. The PILIR was introduced to formalise processes for management of incapacity leave in the public sector workplace, and where necessary, enable employees to be referred for rehabilitation, re-skilling, re-alignment and or retirement^1^. There is a short period of temporary incapacity leave (1–29 days), long periods of temporary incapacity (30 working days or more) and ill-health retirement. The employer is expected to apply for ill-health retirement for an employee “if it is evident that the employee with the impairment or ill-health is not able to RTW following incapacity”^8^,^20^.

When there is no formal disability management (DM) policies, procedures or programme in place, managers use their discretion when monitoring long-term sickness absenteeism due to disability or ill-health^9^. Maja et al.'s^10^ study reported the lack of knowledge about disability by employers which contributes to the poor re-integration of employees with impairments and disabilities within the workplace. The introduction of DM, including integrated workplace rehabilitation and supportive conditions, decreases long-term sick leave and facilitates early RTW^11^.

Disability Management has been defined as a workplace prevention and remediation strategy designed to enable the retention of persons with ill health, injury and acquired disability in employment using a co-ordinated effort with due consideration of the employees, organisation requirements and legal responsibilities^2^,^12^. The utilisation of co-ordinated, cost-effective, rehabilitation services in DM should be considered to facilitate the continual employment of those employees who have experienced functional limitations in the workplace^8^. A focus on the prevention of illness and promotion of healthy workplaces should be included to reduce the incidence of injury, ill-health or acquired disability in the workplace^8^. However, DM cannot occur in isolation and is dependent on other management processes within the organization^8^,^12^. Teamwork, establishing expert interactions within a multidisciplinary team (MDT) including the employer, employee or employee's representative, and DM rehabilitation service providers are essential. There is an increasing need for additional DM rehabilitation service providers to be included in the provision of effective programmes in different workplace environments so that accommodations and RTW services can be managed efficiently and appropriately^8^. Growing awareness of the benefits of DM and the altering financial situation of the competitive universal markets have resulted in organisations developing solutions to return injured workers to the workplace.

The term RTW refers to an assortment of interventions and vocational assessments that are provided following a disabling injury or illness. It is used to describe the duration or extent of the inability to work due to impaired health or functional limitations^13^. A successful RTW system decreases absenteeism rates and enables an employee with ill health or disability to keep or advance inuitable employment within their current workplace. This facilitates the re-integration of the employee into the open labour market and decreases the burden on employers and the economy in terms of disability pensions or incapacity pay-outs^13^.

From a SA perspective, the National Department of Public Service and Administration promulgated the Job Access Strategic Framework in 2006^13^. This framework addresses challenges faced by PWD within the public service with the aim of removing barriers where they exist. Furthermore, the strategic framework, based on social justice principles, aims to ensure that every person enjoys equal opportunity for a better life and for PWD in SA to gain access or maintain their employment opportunities. There are four functional pillars for DM outlined in the Job Access Strategic Framework (2006)^13^, namely, an enabling environment, equality of opportunity (equity), mainstreaming of disability issues, and a barrier-free workplace. The framework describes reasonable accommodation (RA) as “disability specific” and explains that disclosure of disability is a pre-requisite for employees to benefit from accommodations. Issues around disability are sensitive and therefore requires confidential handling within a trustworthy working environment so that issues of disability can be discussed without fear and prejudice. Reasonable accommodations include work place modifications, removal of physical barriers, access to information and technology, equipment and software, adjustment to work schedules, and adjustment to the nature and duration of duties. Reasonable accommodation may be either temporary or permanent depending on the nature and extent of the disability, the job and its essential functions as well as the work environment. It may vary from person to person, even for persons with the same form of disability^13^.

## Disability Management in the Public Health Care Sector

One of the employment sectors most impacted by acquired disability due to occupational injuries, illness and non-communicable diseases is the health care sector. Long-term sickness absence due to disability or ill health has been associated with more significant psychological distress and mental health disorders^14^–^16^. Workplace stressors in a hospital setting such as increased workload, inadequate staffing, organisational problems, and emotional responses to suffering or dying patients add to the anxiety level of health care workers (HCWs)^15^. Stress and burnout are also attributed to the increase in patient loads, having insufficient resources and staff shortages within public health care facilities^15^. Psychiatric illnesses such as depression, stress, anxiety, and burnout are contributing factors to the increase in applications for long-term incapacity leave in the health care sector^11^,^12^. Employees with mental illness often experience prejudice and are at risk of dismissal due to loss of productivity as the result of cognitive limitations, emotional restrictions and poor social interactions^17^.

Health care workers are also prone to musculoskeletal disorders, particularly lower back pain primarily from the incorrect manual lifting of patients^18^. The SA Occupational Health Guidelines^19^ reports chronic diseases such as osteoarthritis, diabetes, hypertension, heart conditions, and degenerative diseases of the back as common conditions affecting the ageing HCW, resulting in long-term absence from work and decreased productivity in the workplace^19^. The principles underlying this guideline^19^ indicate that Occupational Health (OH) and safety legislation must cover workers and employers in all sectors of the economy, and all forms of employment relationships. Moreover, this legislation should prioritise a culture of prevention, have fair compensation and access to rehabilitation, benefit all sectors of the economy, and in all forms of employment relationships. Employers are also expected to carry the cost of accidents and disease in their workplaces, including the cost of medical treatment, compensation and rehabilitation. Employer subsidised OH services which provide health services to employees in the health care sector are limited in the public health care facilities in KZN^15^. A well-managed OH service reduces absenteeism and the use of excessive sick leave. Promoting employee's morale and providing on-site counselling helps to encourage job satisfaction and reduces staff turnover^16^. The OH clinic within the facility that formed the case in this study does not have a DM programme to provide services such as these. The clinic offers medical services for on-duty public healthcare sector staff such as treatment of influenza, headache, emergency medical cases and injury on duty. Part of the services includes medical assessments to facilitate medical boarding of staff who no longer can perform their duties due to physical or mental illness^4^. Additionally, the OH doctor can refer individuals with impairments to the employee assistance programme. The employment assistance programme provides counselling, support or to medical specialists for specialist intervention.

The Public Service Commission explored DM in SA and highlighted limited research on DM in the public sector^1^,^5^. This, together with a lack of the implementation of formal policy guidelines and programmes on DM in the public health care facility, created a need for further exploration in this area. The aim of this study was to therefore explore the DM processes from an organisational perspective at a central hospital in KZN, SA, which operates as a private-public partnership, offering highly specialised medical services to patients in two provinces of the country, and boasting 2315 professional healthcare workers.

## Methods

Use of an explorative qualitative approach^20^ with multiple sources of data as well as locating the study within an interpretive paradigm assisted in an exposition of different stakeholder groups specific experiences, and meanings attached to the issue of DM in the workplace. Purposive sampling was used to select key informants for one focus group and seven semi-structured interviews. A focus group with the nurses was suitable as they were considered a homogenous group. Given the unique roles of the other stakeholders, individual interviews were deemed more appropriate to elicit profession/position specific contributions to the issues around DM.

A focus group was conducted with (n=5) nursing assistant managers who were decision-makers for the six domains that exist within the facility, namely, medical, critical care, paediatric, surgical, pharmacy and allied medical specialities. These managers are responsible for managing employees with disabilities and ill-health and are responsible for the feasibility of implementing RTW strategies within the health care facility. The participants in the interviews (n=7) included a medical manager, a nursing manager, an OH practitioner, an employee assistance practitioner, two senior human resource (HR) officers, and a disability and rehabilitation co-ordinator.

The sessions were conducted in English. Questions were open-ended focusing on different concepts of DM such as definition of Disability and the experience of managing an employee with a disability or ill-health; current DM policies, referral processes, and services offered within the facility, as well as the roles of the various stakeholders. Participants were also asked their views on how the current DM system could be adapted. A moderator was present in the focus group and assisted in recording detailed field notes. Data were digitally audio-recorded and transcribed verbatim and analysed inductively. Initial coding (level one analysis), followed by categorisation of similar codes (level two analysis) ensued. This was followed by pattern matching to identify themes^27^. This process was triangulated by all three authors being involved in the process of data analysis and in ensuring that the themes were an authentic representation of the spoken word of participants. Authors were reflexive in their approach to the analysis and attempted to suspend their judgement (reduce researcher bias) in the process by a reflexive diary and peer-debriefing. Respondent validation occurred at the time of data collection with paraphrasing and summarising at the end of the discussions. Rigour, credibility, and transparency of the process were maintained using three coders and investigator triangulation, a systematic coding process and audit trail^22^. Ethical approval was obtained from the University of KZN Humanities and Social Sciences Ethics Committee (HSS/0168/016M) prior to the commencement of the study in addition to approval from the Provincial Health Ministry. Participation in the study was voluntary, and informed consent was obtained from the participants. This paper only reports on the perspective of the managers and service providers in the DM process.

## Results

The demographics of the participants are presented in Table 1.

**Table 1:** Demographics of Participants (n=12)

		Age	Gender	Occupation	Qualification	Years of experience
**Focus** **Group** **Participants**	1	61	Female	Assistant nurse manager	Bachelor's Degree (Nursing)	32 years
	2	54	Female	Assistant nurse manager	Diploma (Nursing) Bachelors (Technical) Degree in Administration	20 Years
	3	40	Female	Assistant nurse manager	Degree (Nursing) Masters (Nursing Education)	20 Years
	4	60	Female	Assistant nurse manager	Bachelor's Degree (Nursing) Bachelor's Degree (Curriculum)	18 Years
	5	65	Female	Assistant Nurse Manager	Diploma (Nursing) Higher Diploma (Nursing)	23 years
**Key** **Informant** **Interviews**	6	56	Female	Nursing operational manager	Diploma (Nursing) Post-graduate qualification in administration	30 Years
	7	34	Male	Professional nurse-Staff development and Training	Diploma (Nursing) Degree (Nursing)	7 years
	8	35	Female	Medical Officer-OH	Bachelor's Degree (Medical Science)	16 years
	9	44	Female	Social Worker- Employee-Assistance Programme Officer	Bachelor's Degree (Social Work)	26 years
	10	62	Female	Medical Manager	Bachelor's Degree (Medicine & Surgery) Master's Degree (Public Health)	24 years
	11	37	Female	Human Resource Officer	Matriculation (Secondary Education)	16 years
	12	43	Female	Senior Human Resource Officer	National Diploma (Human Resource Management)	22 years

**Figure 1:**
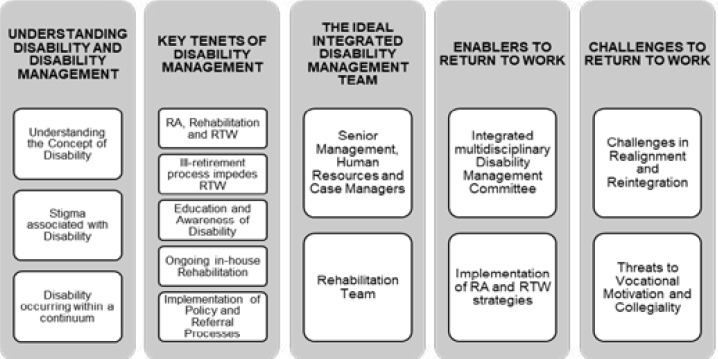
Overview of Themes and Sub-categories that emerged from the data analysis

Five main themes emerged from the data (Figure 1) and are described in Table 2 and augmented by relevant verbatim quotes.

**Table 2:** Emergent Themes from the Study

**UNDERSTANDING DISABILITY AND DISABILITY MANAGEMENT**	
**Understanding the Concept of Disability** A wide range of perceptions was expressed around the concept of disability in the workplace; including the role of the medical officer in the diagnosis of the impairment or disability. Variability in the use of the term disability and impairment was also noted. Participants expressed that an individual must have an impairment and require an assistive device to be functional in a work environment; prior to being considered ‘disabled’. The impact of long-term illness on work performance was also indicated as imperative.	*“PWDS must be diagnosed and treated by a medical practitioner or specialist” **(Participant 2, focus*** ***group participant, assistant nurse manager, female, 54 years)*** *“Illness could be a type of disability especially when they are not fully rehabilitated back to normal healthand* *there is some disability there, that needs to be attended to.” **(Participant 2, focus group*** ***participant, assistant nurse manager, female, 54 years)*** *“PWD, we should not call them disabled, we should call them impaired. A person who is not able to* *perform fully to their capacity because of some problem. Someone that has an impairment that needs* *assistance through a device and that would possibly help him to function in an able-bodied society.”* ***(Participant 6, key informant interview, nursing operational manager, female, 56 years)***
**The stigma associated with disability** The stigma associated with disability and impairment was acknowledged by participants. Perceptions around the capability of impaired or disabled employees were also noted to reflect poor vocational performance and coping, which may not necessarily be a true reflection of employee's abilities.	*I think its because there is that stigma ....for every diagnosis especially with epileptics. It's a big thing ..I* *rather keep quite.....then the next thing is that you see me just falling and all that...”**(Participant 3, focus*** ***group participant, assistant nurse manager, female 40 years)*** *Judgemental, some people judge you, other people feel they cannot render the service as they used too.* *Now they feel inferior. The lack of understanding of disability as well. So we just assumed that if a person* *has an illness they can't come back to work...**(Participant 4, focus group participant, assistant nurse*** ***manager, female 60 years)***
**Disability occurring within a Continuum** Disability was considered to occur within a continuum with variable presentation and degree of impairment. With this understanding, participants acknowledged the need for accommodation as employees may have residual skill and abilities.	*“So, you might have total disability, partial disability and then based on that try and see what they exactly* *they can do. E.g. if someone maybe physically disabled with their legs can still can use their arms, they* *can be accommodated. With any impairment, be it sight, you know, physical, mental. Even mental you* *can have impairment or disability.” **(Participant 3, focus group participant, assistant nurse manager,*** ***female, 40 years)*** *“it can be physical; it can be emotional because you can have disability of the mind so if they not able to* *perform fully per maximum capacity then there is some disability which needs to be attended to.”* ***(Participant 2, focus group participant, assistant nurse manager, female, 54 years)***
**KEY TENETS OF DISABILITY MANAGEMENT**	
**RA, Rehabilitation and RTW** The participants indicated that a DM programme should offer the provision of assistive devices, RA, education and awareness and rehabilitation to facilitate early RTW and to improve productivity in the workplace. All participants reported that these inclusions are essential to retaining staff with ill health and disability. The RTW strategies such as flexible work hours, job re-alignment and modification of duties were reported to have been implemented.	”It revolves around assistive devices, reasonable accommodation, education and awareness and rehabilitation as well. So, if you consider all those four concepts it would encapsulate disability management. If it is managed according to those four pillars.” ***(Participant 7, key informant interview,*** ***staff development and training nurse, male, 34 years)*** “...to help the individuals to work independently, if you work independently other than having someone to assist you with the task like. if you are short and you can't reach the top we need to provide you with a device like a stool or step. If you have short sight then we need to arrange the computers with a program. I think it helps because it helps the person to be independent he can't wait for somebody else to do the task for him. Very helpful ***(Participant 2, focus group participant, assistant nurse manager,*** ***female, 54 years)*** “Well reasonable accommodation is basically adapting, adapting things to suit the person affected with a ”It revolves around assistive devices, reasonable accommodation, education and awareness and rehabilitation as well. So, if you consider all those four concepts it would encapsulate disability management. If it is managed according to those four pillars.” ***(Participant 7, key informant interview,*** ***staff development and training nurse, male, 34 years)*** “...to help the individuals to work independently, if you work independently other than having someone to assist you with the task like. if you are short and you can't reach the top we need to provide you with a device like a stool or step. If you have short sight then we need to arrange the computers with a program. I think it helps because it helps the person to be independent he can't wait for somebody else to do the task for him. Very helpful ***(Participant 2, focus group participant, assistant nurse manager,*** ***female, 54 years)*** “Well reasonable accommodation is basically adapting, adapting things to suit the person affected with a disability. Adapting, modification areas. So, reasonable accommodation is basically, modifications and adaptations of the work area.” ***(Participant 8, key informant interview, medical officer - occupational*** ***health doctor, female, 35 years)*** “I think reasonable accommodation is where there are accommodations in the workplace to the extent where they can still use their professional ability. So, if it is a professional nurse and her job is to do vitals, injections, lifting and whatever it is, so basically after being disabled, you need to assess what they can do. But it must fit in with their profession.” ***(Participant 4, focus group participant, assistant*** ***nurse manager, female, 60 years)***
**Ill-Retirement Process impedes RTW** The overall ill-health retirement process at the healthcare facility was viewed to impede the RTW process. The committee appears to have authority to make a decision around ill-health retirement based on their judgement even if this may not have been requested. These processes impact on the employment status of the employee and have financial implications.	“The current ill health retirement process looks at things too superficially ok and again it's a very long drawn out process and doesn't always accept the boarding applications also the financial implications to these patients are another issue. Some instances they don't apply for ill health retirement but the committee recommends it if they see you have been away for too long, they call you in and assess you because they have their own panel of specialists, and if they feel you won't recover then they will recommend it” ***(Participant 8, key informant interview, occupational health doctor, medical officer -*** ***occupational health doctor, female, 35 years)***
**Education and Awareness of Disability** Participants highlighted that education and awareness of disability lacked within the facility. Education for the supervisor, employees and management was indicated as necessary with a focus on teamwork and management of the person with the impairment in the workplace.	Education that you give to the staff and manager as well. Firstly, we should initiate a disability management programme in the institution quite extensively in the understanding of disability. In terms of the DM system in the institution, we need to have it central. The people need to understand what needs to happen when I do fall I'll know what to do and not wonder where do I go.'*’ **(Participant 8, key*** ***informant interview, medical officer - occupational health doctor, female, 35 years)***
**Ongoing-in-house Rehabilitation** Ongoing in-house rehabilitation and re-integration were lacking. Ongoing rehabilitation in terms of occupational therapy, physiotherapy and psychological services and RTW programmes should be conducted in house reducing absenteeism from work. The employees have the option to consult with in house specialists or seek consultation from outside specialists. These delays could result in the person with the impairment needing to apply for long-term incapacity leave or application for medical board rather than being accommodated in the workplace.	“The rehabilitation programme should be more intensive. I don't know whether it may sound too first world. May be getting the team to take the person to their workplace, observe on the job or see what can be done maybe the technique of holding some thing or the way the client is bending or lifting a patient.” ***(Participant 2, focus group participant, assistant nurse manager, female, 54 years)*** The policy is linked to DPSA policy....5...there is no formal process.... we just use our discretion. ***(Participant 10, key informant interview, medical manager, female, 62 years)*** “The manager needs to manage disability within workplace. Using the different management styles but focusing on re-integration, multidisciplinary teamwork and understanding the impacts of reasonable accommodation and assistive devices together with education” ***(Participant 6, key informant interview,*** ***nursing operations manager, female, 56 years).*** “The role of the immediate line manager is to identity that there is problem....it can be an illness especially the long-term ... to is a problem this needs attention...creating an environment where the workers also feel that they can come and they can talk to the manager... open and transparent system of management of ill health and disability” ***(Participant 6, nursing operations manager, female, 56*** ***years).*** “The supervisor needs to contact our Occupational health clinic and the doctor will do the fitness assessment to check the disability” ***(Participant 9, key informant interview, social worker-employee*** ***assistance programme practitioner, female, 44 years)*** “In terms of the overall employee health we have got the EAP system so if there are, and we got the occupational health in terms of screening and making sure that employees are cared for. The EAP for support and counselling and preventative programme.” ***(Participant 10, key informant interview,*** ***medical manager, female, 62 years)*** “Your starting point will be your occupational health clinic then obviously. we all have a right to choose our medical practitioners some of them maybe outside practitioner but everything needs to be referred to occupational health clinic in house and from there I would think if the doctors, need then any further assistance they would then refer to the rehabilitation specialists.” ***(Participant 8, key informant*** ***interview, occupational health doctor, medical officer - occupational health doctor, female, 35*** ***years)***
**Implementation of Policy and Referral Processes** Managers stated that the integrated national policy on disabled people in South Africa is the only policy they are aware of and no other policy is available on DM at the facility. There are no structured referral processes, and managers use their discretion when managing the person with an impairment or ill health.	“There is one policy that is the DOH policy that deals with disability at the workplace but that is general disability....err.... That deals with Recruitment and selection ... Nothing deals with rehabilitation or reintegration back into the workplace. There is no policy of that nature.” ***(Participant 11, key informant*** ***interview, human resource officer, female, 37 years)*** “No structured referral pathway and they come too late I think because they have the option of going too private and coming here they sort off delay. Then they end up in ill health retirement whereas they could have come earlier on, they would have been accommodated in the workplace and not being off sick or awaiting medical boarding.” ***(Participant 8, key informant interview, medical officer - occupational***
**Rehabilitation Team** The rehabilitation team should consist of physiotherapists, speech therapists, psychologists and occupational therapists. The participants indicated that occupational therapy plays a significant role in DM through their knowledge and understanding of disability, function, RA, and provision of assistive devices. They are also essential in providing recommendations in terms of RTW strategies.	“We work with physiotherapy, occupational therapy and other rehabilitation specialists if there is a need like psychologists, speech therapists if cognitive problems. Psychologist, occupational therapist or the professional team and of course will include the social worker if there are social issues involved.” ***(Participant 5, focus group participant, assistant nurse manager, female, 65 years)*** “The occupational therapists has an important role in DM. She understands disability the best, her role in in rehabilitation and understanding the aspects of RA and how to apply assistive devices to certain disabilities is of utmost importance. And I think medical practitioners and occupational health doctors, and physiotherapy lack that so in terms of that an occupational therapist will be key in ensuring the reintegration from a scientific process. Occupational therapy understands the different disabilities and the needs of the different disabilities and how to apply assistive devices using the assistive devices to enhance productivity and with RA.” ***(Participant 7, key informant interview, staff development and*** ***training nurse, male, 34 years)***
**ENABLERS TO RTW**	
**Integrated multidisciplinary DM Committee** Participants emphasised the need for an integrated, multidisciplinary DM committee. The committee should formulate integrated DM institutional policies and procedures to monitor ill health and disability within the workplace. Additionally, education should be conducted on several levels, e.g. managers should be educated on DM, co-worker education is required, and the person with the impairment require knowledge on how to manage their disability.	“I think the big enabler is to have a policy on comprehensive occupational health for DOH employee that is well resourced because we don't have that. We need to have a well-resourced team by the resources I mean the numbers. We have the expertise but we falling short because of the numbers. As I said that it is something that is very under developed and we need to put more energy and effort into making sure that it is well established. The focus is on health and safety is only one aspect.” ***(Participant 10, key*** ***informant interview, medical manager, female, 62 years)*** “I think firstly we need to have a system that accommodates the employees of being sick or disabled. We need to utilise our in house staff. We need to have flexible hours and adapt jobs so the person can RTW.” ***(Participant 4, focus group participant, assistant nurse manager, female, 60 years)***
**Implementation of RA and RTW Strategies** The participants reported that all measures of RA should be implemented, as this would improve productivity at the facility. Implementation of RTW strategies such as flexible work schedules and re-alignment of duties is essential to facilitate re-integration into the workplace. The participants reported that the manager plays a key role in creating a conducive environment for work. Acceptance from co-workers and managers were considered crucial factors facilitating successful early RTW.	“I think the key issue is about the manager's role of creating a conducive environment you know where the person feels that they can come back to full function.” ***(Participant 9, key informant interview,*** ***social worker-employee assistance programme practitioner, female, 44 years)*** “Acceptance from the other staff, colleagues are also important because it creates a conducive and accepting environment of the employee to RTW following illness or disability.” ***(Participant 3, focus*** ***group participant, assistant nurse manager, female, 40 years)***
**CHALLENGES TO RTW IN THE WORKPLACE**	
**Challenges in Re-alignment and Reintegration** Participants reported that employees do not want to be reallocated due to the financial implications of losing their occupation-specific dispensation (OSD) for working in a specialised unit like intensive care. Managers reported that it is difficult to accommodate a specialist nurse in critical care units because there are no light duties in nursing. Participants reported there is an absence of formal policies and procedures, and they use their discretion and informal processes when dealing with an employee with ill health or disability.	“There is this OSD. Its regarding money while working someone has been diagnosed with a disability and is unable to work on a patient it is difficult to move them out of that area, once you remove them from there, apparently their OSD is supposed to be stopped.” ***(Participant 5, focus group participant,*** ***assistant nurse manager, female, 65 years)***
**Threats to vocational motivation and collegiality** HCW are, however, still regarded as a fulltime member of staff despite being allocated a form of light-duty and not being involved in the clinical care of the patient. It is frustrating for the employee with the impairment as well as other staff because they are unable to execute the allocated work or the assistive devices they require are not available.	“I think it is the sheer workload in relation to the staff load. The attitude of staff but in my opinion I think it is the shear workload and attitude. There is no light duties often.” ***(Participant 10, key informant*** ***interview, medical manager, male, 62 years)*** “There is no re-integration process in place to assists a PWD [ person with an impairment] or Ill-health it frustrates the employees because they can't do their work and we don't have proper devices for the people e.g. we have another staff that requires a motorised wheelchair but we don't have it in the hospital specifically for her needs.” ***(Participant 2, focus group participant, assistant nurse manager,*** ***female, 54 years)***

## Discussion

Twenty-five years into democracy in SA, the perception remains that individuals with impairments and disabilities are discriminated against in terms of their right to work. This is despite the introduction of the PILIR in 2005 in SA, which guides the management of incapacity leave in the public health sector^1^, the Job Access Strategic Framework (2006)^13^ and the Employment Equity Act of 2008^2^. These legal treaties espouse the rights of the employee with a disability or ill-health in South Africa and outline processes for accommodating the RTW of employees with an acquired disability or ill-health. However, there is limited implementation of these policies and laws. There is support in available literature for a review of institutional workplace disability policies, which will allow for equitable employment and retention of employees with ill health, injuries and acquired disability within the public health care service^1^,^21^,^22^.

In this study, factors that posed as barriers to implementing DM processes within the workplace were highlighted. Disability Management was not clearly understood by senior management and OH practitioners, with managers reporting a lack of understanding around the definitions of employees with impairments and disability, necessitating education and awareness around these two concepts. This finding was similar to that of a project that evaluated the impact of PILIR on sick leave trends in the public service^1^. Moreover, there appeared to be a limited understanding of PILIR process that focussed on the retention of employees with impairments and disabilities in the workplace.

Stigma associated with disability and impairment was also reported and negatively influenced the DM and RTW process. Maja and colleagues^10^ in their SA study also found that managers were not always aware of the challenges at the ground level which inevitably impacted on the accommodations which were made for the employees. Discrimination and stigma were also noted^10^.

Another challenge to DM related to re-alignment and re-integration. There is often no alternative task for that cadre of a worker to engage in especially with specialised employees. A lack of formal policies and procedures as well as appropriative assistive devices to aid employees with a disability or those with ill-health were noted in this study. This fragmented DM policy led to managers having to make decisions for employees who became ill or were injured at work. The need for an integrated OH and DM policy focusing on holistic early RTW and re-integration of the employee also emerged as essential within the workplace, in keeping with a study by Haafkens^23^ which emphasises the vital role line managers, and supervisors play in workplace health and early RTW.

A lack of services, including rehabilitation for the employee with the impairment and the management of disability and illness in the institution was highlighted. The application process for long-term incapacity leave and ill health retirement was seen as ambiguous, tedious and challenging, and marked with financial implications for the employees and was perceived to hinder an effective DM process. These findings were similar to other studies^1^,^9^.

Despite the existence of numerous RA policies and RTW policies in SA, there is a lack of implementation and monitoring of these policies within the health care setting. These can be attributed to a lack of education and awareness programmes, limited human resources and inadequate formal DM process and programmes within the public health care sector^8^. Perhaps, the lack of litigation from an employee against the public sector has resulted in less stringent monitoring or implementation of RA or RTW. In this study, it emerged that participants felt there is a need for comprehensive disability awareness programmes that need to be directed at all stakeholders in the institution, which includes managers, co-workers and employees with impairments and disability. This is supported by Majola and Dhunpath in their study^21^, in which disability awareness was noted as a crucial function of all levels of management if DM was to be adequately provided within the work setting.

Notwithstanding these, the participants in this study highlighted enabling factors for RTW. These included the provision of assistive devices, RA such as re-alignment of duties or reallocation to other departments or having a flexible schedule. This inclusion would assist in retaining staff with ill-health. Additionally, having a supportive, caring and empathetic supervisor, and co-worker support, created a conducive work environment for RTW. A need for ongoing in-house rehabilitation to aid in RTW programmes and to assist with reducing absenteeism was also noted.

In this study, formal processes and policies for referral and management of PWD within the facility were highlighted as necessary for a successful DM process. Hospital senior management being willing to participate in the DM process is crucial. The hospital manager was perceived to create the organisational culture and beliefs toward the DM process especially with regard to emotional support, environmental support in terms of the implementation of RA measures like removal of physical barriers and provision of workplace assistive devices. Current practices at the institution in this study appeared to focus on prevention and promotive programmes, including an employee assistance programme and OH services but lacked rehabilitation and re-integration programmes. The HR managers are responsible for developing organisational policies and in providing advice or guidelines on labour issues, for the management of the employee with impairment or disability in the workplace. This includes personnel management in terms of safety and wellness of the employee, administrative procedures and management of disability benefits. The need for transparent communication between HR and managers in this study highlighted as a critical aspect to ensuring effective DM process.

Additionally, HR should ensure that disciplinary measures are implemented for those employees who are found to exaggerate symptoms and abuse the long-term incapacity leave process. Studies^22^,^23^ indicate that more work needs to be done to change attitudes about disability, especially within the public sector. Disabled people of South Africa (DPSA)^22^ reported that workplace profiling is essential for organisations for proper incentives to be considered for retention of PWD as well as for the employer to be able to meet the necessary RA needs.

All managers in this study emphasised that an integrated DM committee was the way forward in terms of developing a consistent DM process and practices. The DM committee should comprise of OH, HR, employee assistance practitioner, senior management team, physiotherapist, occupational therapist, psychologist and social worker. This is in keeping with Schreuer and colleagues^24^ who view DM as a MDT approach involving multiple role players, including, employees, management, OH doctor or nurse, supervisor, HR administrator, physicians, the rehabilitation team, union representative, employee assistance practitioner, middle management, senior management and job coaches. Each one of these team members has a specific role in DM for the programme to be successful in the workplace^8^. Managers in this study emphasised the need for additional HR dedicated towards DM for employees within the workplace and the need for the rehabilitation team, to act as key role players in facilitating early RTW strategies for successful DM. This is in keeping with the findings on the evaluation of the impact of PILIR on sick leave trends in the public service^8^. Additionally, there needs to be a rehabilitation team that should consist of an occupational therapist, physiotherapist, speech therapists and psychologist. This rehabilitation team, particularly the occupational therapists, would play an essential role in providing return to work recommendations and RTW strategies and assist with job coaching. In their paper, Govender and colleagues^9^ outlined the role that the occupational therapist can play in medical incapacity management in South Africa. Given the many factors that impact an employee's health, a proactive approach towards preventing illness and limiting disabilities for the SA workforce is required^25^. DM should be used as a tool to redress disability issues and reasonably accommodate PWDs within the workplace, thereby enhancing diversity management and increasing productivity in the workplace^23^.

## Conclusion

This study has created insight into how the DM process is implemented and the barriers, facilitators and a possible way forward in a single site. Barriers include poor adherence to OH and DM policies with informal referral processes. Managers using discretion to manage employees with ill-health remains the status quo in the organisation included in this study. A lack of education on definitions of employees with impairments and disability together with a lack of awareness of rehabilitation programmes were highlighted as an obstacle to the DM process. Participants in this study perceived that the enabling factors for RTW should include a supportive, caring and empathetic supervisor, teamwork, co-worker support, and re-alignment of duties or reallocation to other departments, flexible work schedules, positive attitudes of the employee involved and a conducive work environment. The way forward includes a need for an integrated multidisciplinary DM committee, more cohesive DM polices and referral processes and education for the supervisor, employees and management as necessary with a focus on teamwork and management of the person with the impairment in the workplace. Additionally, there was a need for ongoing rehabilitation (occupational therapy and physiotherapy) and psychological services, which would facilitate RTW programmes to be conducted in-house, thereby reducing absenteeism from work. The authors' believe that the initial increase in financial outlay to sensitise managers to a streamlined DM process and to implement these rehabilitation services, would be recompensed through the reduction in work days lost to absenteeism and staff shortages, as employees with disabilities or illhealth would be accommodated within in the workplace.
